# Cardiovascular Collapse During Scoliosis Surgery in a Patient With Coffin-Lowry Syndrome and Mesocardia

**DOI:** 10.7759/cureus.94769

**Published:** 2025-10-17

**Authors:** Tara Ali, Abdulrahman Albarni, Michel Guez, Josefin Åkerstedt

**Affiliations:** 1 Department of Anesthesiology and Intensive Care, Umeå University Hospital, Umeå, SWE; 2 Department of Diagnostics and Intervention, Umeå University, Umeå University Hospital, Umeå, SWE; 3 Department of Orthopedics, Spine Unit, Umeå University Hospital, Umeå, SWE

**Keywords:** anesthesiology, cardiovascular collapse, coffin-lowry syndrome, corrective spine deformity surgery, mesocardia, posterior spinal fusion, scoliosis, spine surgery

## Abstract

Coffin-Lowry syndrome (CLS) is a rare X-linked disorder marked by intellectual disability, craniofacial and skeletal anomalies, and progressive spinal deformities like scoliosis and hyperlordosis. Corrective posterior spinal fusion (PSF) surgery is often required. Mesocardia, a congenital anomaly where the heart is positioned centrally in the thorax, may affect perioperative hemodynamics, particularly in the prone surgical position. This report presents a case illustrating the perioperative challenges of PSF in a patient with both CLS and mesocardia.

A 17-year-old male with CLS, mesocardia, and severe scoliosis underwent PSF (T2-S2). During prone positioning, the patient experienced a gradual hemodynamic collapse, characterized by a decrease in oxygen saturation and a marked drop in both pulse rate and blood pressure. Repositioning to the supine position led to immediate recovery. Imaging ruled out pulmonary or vascular obstruction, pneumothorax, and embolism. The event was attributed to mechanical cardiac compression related to syndromic soft tissue laxity, thoracic deformity, mesocardia, and posterior soft-tissue release. Surgery was completed using a modified lateral oblique prone position, maintaining hemodynamic stability.

This case illustrates the importance of anticipating cardiopulmonary compromise due to anatomical and positional factors and that personalized surgical planning and modified positioning can be crucial in complex spinal deformity surgery in syndromic patients with thoracic abnormalities.

## Introduction

Coffin-Lowry syndrome (CLS) is a rare X-linked disorder affecting approximately one in 50,000 to one in 100,000 individuals, characterized by intellectual disability, distinctive craniofacial features, heart problems, hearing loss, seizures with sudden loss of muscle tone, and skeletal abnormalities, including progressive spinal deformity that often worsens in adolescence [1,2]. In this age group, posterior spinal fusion (PSF) is commonly employed for definitive scoliosis correction [3]. Mesocardia, a congenital cardiac malposition in which the heart lies centrally within the thorax with the apex directed midline (neither left-sided/levocardia nor right-sided/dextrocardia), typically occurs without situs inversus and is usually asymptomatic, and is rare with an estimated prevalence of approximately 0.2% [4,5]. In the presence of thoracic deformity, mesocardia may further accentuate intraoperative hemodynamic vulnerability.

Prone positioning is known to influence cardiovascular physiology by increasing central venous pressure and systemic vascular resistance while decreasing venous return and cardiac output [6-8]. In patients with mediastinal mass, morbid obesity, or severe thoracic deformity, these effects may be amplified by direct compression of the heart or great vessels, leading to pulmonary artery hypertension and reduced preload [9-11]. Understanding these mechanisms is critical when evaluating syndromic patients with restricted thoracic space or cardiac displacement, as they may be particularly prone to cardiovascular instability during prone spinal surgery. Because intraoperative risks in syndromic scoliosis with coexisting cardiac or mediastinal anomalies are sparsely described in the literature, we present the following case.

## Case presentation

A 17-year-old male with Coffin-Lowry syndrome presented with characteristic phenotypic features and complex disabilities, including profound intellectual impairment and stimulus-induced drop episodes. He was non-verbal and had previously demonstrated high functional mobility during early adolescence, engaging in activities such as skiing and skating. This level of mobility had declined markedly in recent years due to progressive scoliosis, and at presentation, he was ambulatory only with the assistance of a walker.

Comorbidities included mild aortic insufficiency, which remained stable on serial echocardiography, and mild asthma. Cardiology follow-up demonstrated preserved ventricular function with mild aortic valve regurgitation, normal blood pressure, and a normal transthoracic echocardiogram (TTE). Preoperative chest computed tomography (CT) confirmed the presence of mesocardia.

**Figure 1:**
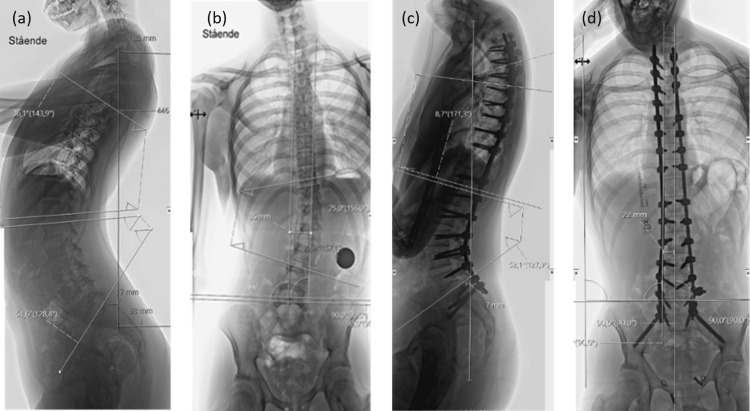
Preoperative standing radiology lateral (a) and AP (b) spine X-rays showing scoliosis and hyperlordosis. Postoperative standing radiology lateral (c) and AP (d) spine X-rays.

Radiographic evaluation revealed a rapidly progressive left-convex thoracolumbar scoliosis between T11 and L3 measuring 25°, thoracic lordosis of 36° (T5-T12) and 25° (T2-T12), and lumbar lordosis of 50° (L1-L5). The sagittal vertical axis (SVA) measured -17 mm, accompanied by a coronal trunk shift of 35 mm, resulting in complete loss of both sagittal and coronal balance (Figures 1a, 1b). Whole-spine magnetic resonance imaging (MRI) revealed no abnormalities such as syringomyelia, tethered cord, or spinal cord compression. In view of the documented curve progression, posterior spinal fusion (PSF) with deformity correction was planned. The patient was admitted for surgery in good general condition.

Anesthetic and surgical course

Anesthesia was induced using total intravenous anesthesia (TIVA) with propofol, remifentanil, and esketamine. Intubation was achieved at approximately 8:15 a.m., via video laryngoscopy without difficulty. To permit reliable intraoperative neurophysiological monitoring (IONM), Somatosensory Evoked Potentials (SSEPs) and Motor Evoked Potentials (MEPs) were continuously recorded during surgery, and a single dose of rocuronium (0.6 mg/kg body weight) was given during intubation. Additional intraoperative monitoring included TTE, radial artery line, central venous access, urinary output, and cell salvage. A 500 ml crystalloid bolus was administered at the start of surgery, followed by a maintenance infusion of crystalloids at 2-3 ml/kg body weight/h throughout the procedure. The patient did not require vasopressors, although a routine norepinephrine infusion (0.01-0.02 µg/kg body weight/min) was maintained. The total volume of crystalloids administered was 3390 ml. The diuresis was approximately 102 ml/h.

**Figure 2:**
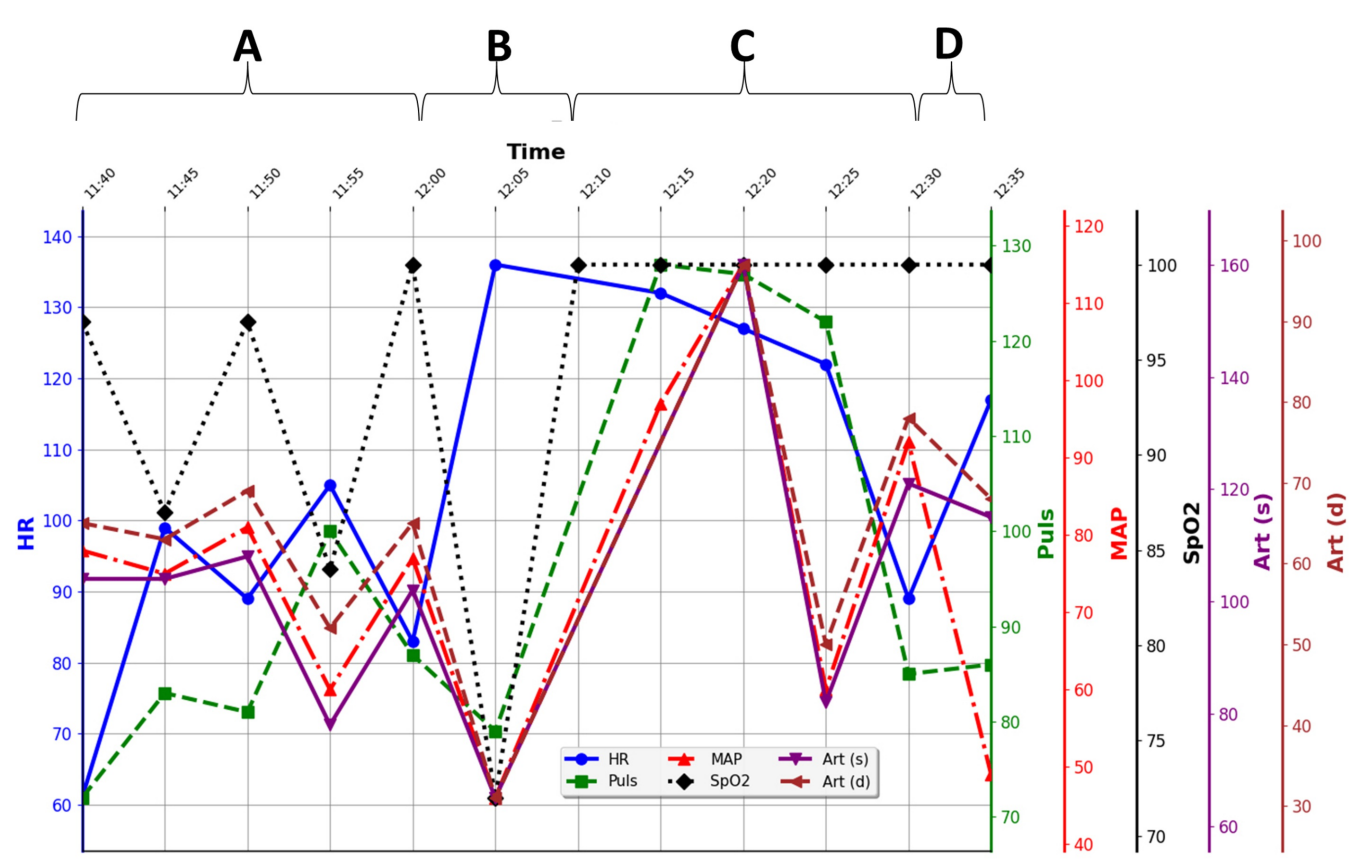
Intraoperative parameters during the cause of hemodynamic instability. (A) Gradual decrease in oxygen saturation during surgical dissection and exposure in prone position. (B) Critical desaturation and hypotension. (C) After repositioning in supine position. (D) Recurrent instability upon repositioning in prone position. Pulse is measured in beats per minute (bpm), mean arterial pressure (MAP), systolic (Art(s)) and diastolic (Art(d)) blood pressure in millimeters of mercury (mmHg), and saturation (SpO2) in percent (%).

PSF with instrumentation from T2 to S2, without osteotomies, was planned. The patient was positioned in the prone position on a Jackson spine table. Prone positioning commenced at 9:06 a.m. Initial exposure and screw placement (L2-S2), with navigation-assisted placement confirmed by intraoperative computer tomography, commenced around 10:20 a.m. and proceeded without complication. During the extended dissection from L2 to T3, the patient's oxygen saturation decreased gradually (Figure 2, event A), even though the end tidal carbon dioxide (EtCO_2_) remained normal. Intraoperative ventilatory parameters, including exhaled tidal volume, respiratory rate, and peak inspiratory pressure, are presented in Appendix 1. These data demonstrate a slight transient increase in peak inspiratory pressure at the onset of desaturation, while end-tidal CO_2_ remained within normal limits. Manual breathing assistance and an increased fraction of inspired oxygen (FiO_2_) were administered. To rule out airway obstruction, bronchoscopy was performed in the prone position, which showed no secretion, airflow obstruction, or endotracheal tube kinking. The right main bronchus was visualized with a normal contour, whereas the left main bronchus, which is normally narrower, appeared mildly flattened. No obstruction was seen in either bronchus. At the time of bronchoscopy, the patient had already begun to desaturate (Figure 2, event A). Bronchoscopy was initiated at 11:50 and lasted less than two minutes. As shown in Appendix 1, there was no evidence of air trapping or auto-PEEP during the procedure. The patient was intubated with a 7.0 mm internal diameter endotracheal tube (Rush), and bronchoscopy was performed using an Ambu aScope 4 Broncho Slim (Ambu A/S, Ballerup, Denmark) with an outer diameter of 3.8 mm and an inner diameter of 1.2 mm. This size combination provided adequate airflow around the bronchoscope, minimizing the risk of air trapping or hemodynamic compromise during the brief examination.

Upon further evaluation, the patient experienced a hemodynamic collapse, with further desaturation to approximately 70%, profound hypotension (65/31 mmHg), and a marked drop in pulse rate around 43 bpm (Figure 2, event B). The patient was turned to the supine position, and cardiopulmonary resuscitation with cardiac compressions was initiated immediately, resulting in instant stabilization and restoration of normal hemodynamic parameters (Figure 2, event C). Bedside TTE showed good global and regional wall motion and size, a mild increase in regurgitation at the aortic valve, and mild hypovolemia. The patient was treated with crystalloid (approximately 30 ml/kg body weight) and albumin infusion (250 ml infusion with 50 g/l albumin), along with a single dose of atropine (0.5 mg) (Figure 2, event B). After stabilization in the supine position, repositioning to the prone position was attempted, but triggered hemodynamic instability once again (Figure 2, event D). The wound was therefore temporarily closed in the lateral position, and the patient was transferred to the ICU for further work-up, including urgent CT angiography, which ruled out pulmonary embolism and excluded other acquired cardiac or pulmonary abnormalities.

Management and outcome

**Figure 3:**
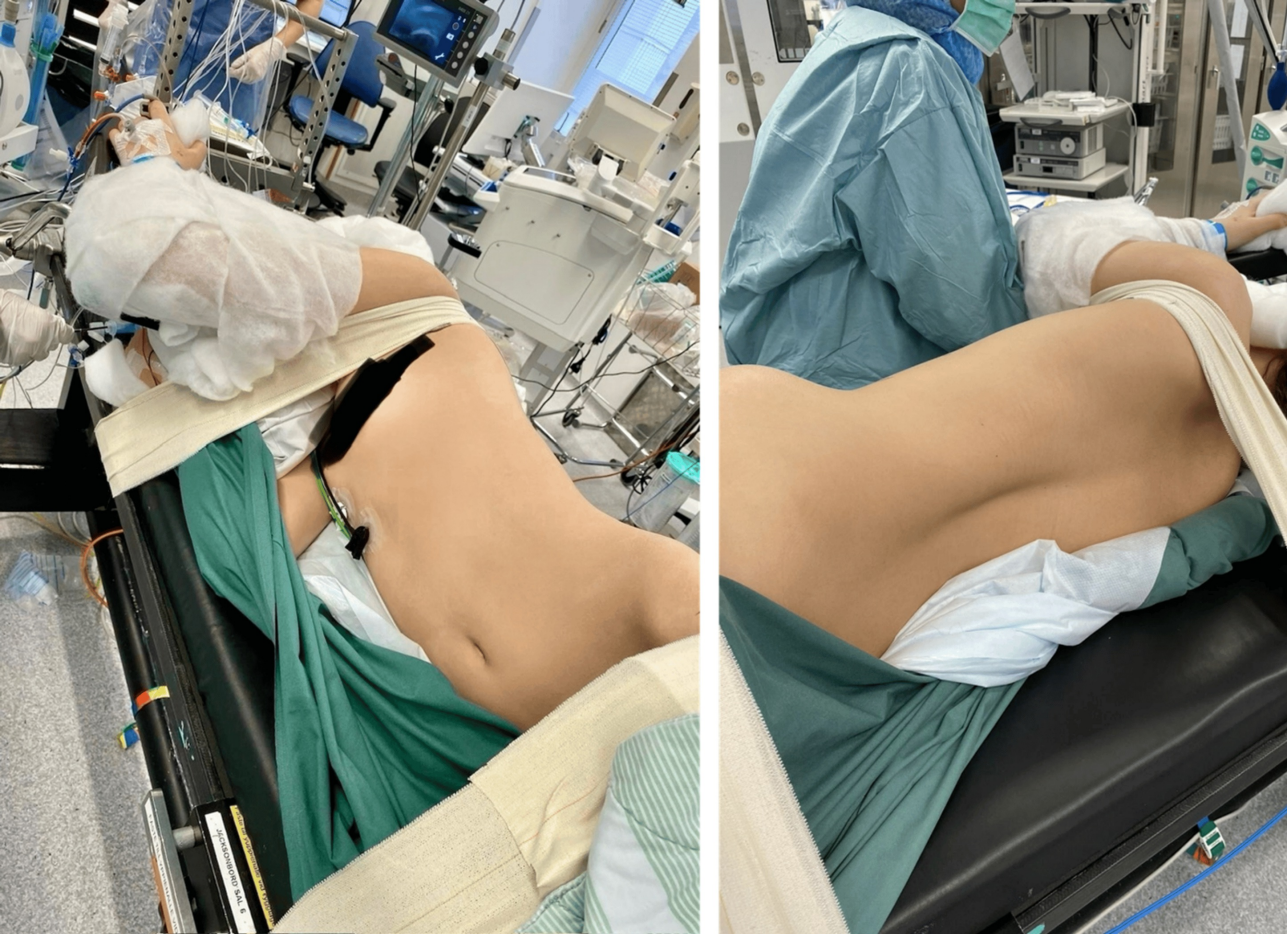
Positioning in the right lateral oblique-prone position (right side down, left side up). The individual shown is not the actual patient from the case but demonstrates the same positioning used during surgery.

The following day, the patient was deemed stable and returned to the operating room for staged completion of surgery. He was placed in a right lateral oblique-prone position (Figure 3), optimizing access and cardiovascular stability.

In the right lateral oblique position, screws were placed fully navigated unilaterally (left pedicles) from T3-L1 and fixed with a temporary rod. The patient was then repositioned to the prone position, which was well tolerated. Hemodynamic parameters remained stable in the prone position throughout the remainder of the procedure. Final correction was performed in the prone position, the remaining levels were instrumented, and kyphosis correction between T3-T10 was achieved using global rod derotation, sequential tightening, distraction, and in situ rod contouring techniques with cobalt-chrome rods. The preoperative left-convex thoracolumbar scoliosis was corrected from 25° to 3° between T11-L3, thoracic lordosis from 37° to a thoracic kyphosis of 9° (T5-T12). The preoperative lumbar lordosis remained unchanged at 50° (L1-L5) postoperatively. The sagittal vertical axis (SVA) was negative 17 mm before surgery and corrected to negative 7 mm after surgery. The preoperative significant coronal trunk shift of 35 mm was corrected to 20 mm. The overall radiological improvement in sagittal balance and alignment is shown in Figures 1a-1d.

IONM remained stable throughout surgery. The estimated blood loss from the initial surgeries was approximately 1000 ml, and from the second surgery, approximately 500 ml. Postoperative imaging confirmed satisfactory alignment in both coronal and sagittal planes. Some residual lumbar lordosis was accepted due to anatomical constraints and sacral orientation (Figures 1c, 1d). Postoperative rehabilitation was uneventful, and the patient showed good recovery at the three-month follow-up.

## Discussion

This case highlights the rare but potentially life-threatening hemodynamic instability that may occur in patients with complex syndromic spinal deformities undergoing PSF. In this patient, the combination of CLS, severe scoliosis with thoracolumbar hyperlordosis, and mesocardia likely contributed to intraoperative cardiovascular collapse in the prone position.

During dissection from L2 to T3, oxygen saturation gradually declined, while end-tidal CO_2_ remained normal, thereby reducing the likelihood of pulmonary embolism as the underlying cause. Intraoperative bronchoscopy in the prone position ruled out secretion or obstruction; however, the left main bronchus appeared more flattened than expected, suggesting possible mild extrinsic compression.

In accordance with previous reports, prone positioning in patients with combined spinal and chest-wall deformities can result in obstructive hypotension caused by mechanical cardiac or great-vessel compression rather than by anesthetic depth or hypovolemia. Pediatric scoliosis in association with pectus excavatum may demonstrate abrupt and occasionally refractory hypotension upon turning to the prone position, often resolving with repositioning [6-8]. Similarly, patients with severe thoracic lordoscoliosis possess limited cardiopulmonary reserve [9]. Abcejo et al. (2017) directly demonstrated such positioning-dependent compression using transesophageal echocardiography in a patient with scoliosis, showing collapse of the left atrium and biventricular failure, consistent with direct cardiac compression impairing both filling and pumping function [10]. In the present case, the limited intrathoracic space is likely to predispose to further compression once in the prone position. The hemodynamic deterioration observed during surgical manipulation suggests a dynamic, pressure-induced component, analogous to a mediastinal mass effect, where transient external pressure on the sternum and anterior chest wall further exacerbated cardiac compression.

Prone positioning typically reduces cardiac output through decreased venous return, altered arterial resistance, or reduced left ventricular compliance [11]. This effect may be amplified by abdominal compression, which can elevate epidural venous pressure and increase the risk of bleeding [12]. Allowing the abdomen to hang freely, as with the Jackson table used in this case (Appendix 2), helps mitigate these effects by minimizing intra-abdominal pressure and improving venous return. In a randomized echocardiographic study, Dharmavaram et al. (2006) demonstrated that positioning on several different spine tables can significantly reduce cardiac output, cardiac index, and stroke volume, likely owing to impaired preload or elevated thoracic pressure [13]. These observations underscore the importance of meticulous intraoperative positioning and hemodynamic monitoring, particularly in patients with compromised cardiac reserve [12,13].

Neuromuscular deformities are associated with an increased risk of intraoperative bleeding, although the mechanisms underlying this tendency are not yet fully understood [14,15]. In the present case, a total blood loss of 1,500 ml was considered moderate, given the two-stage procedure, and was effectively compensated by a balanced transfusion strategy comprising 231 ml of autologous blood via cell saver, two units of erythrocyte concentrate, 500 ml of 5% albumin, one unit of plasma, two grams of fibrinogen, and maintenance crystalloids. The choice of blood products and fluids was guided by rotational thromboelastometry (ROTEM), a coagulation-specific assessment. Tranexamic acid (1 mg/kg body weight) was administered as a preoperative bolus, followed by a continuous infusion of 1 mg/kg body weight/h until wound closure, in accordance with standard practice in pediatric scoliosis surgery [16-18].

**Figure 4:**
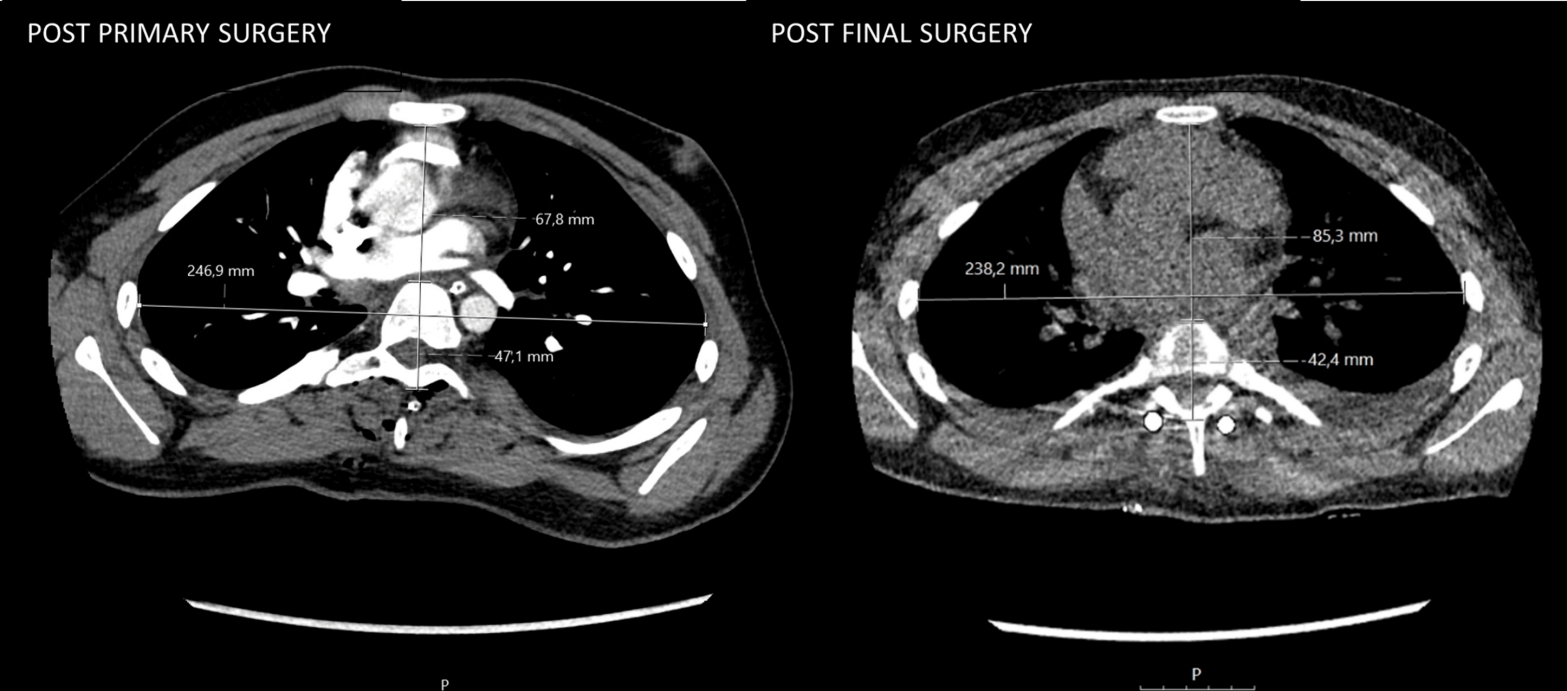
After initial surgery (left) and post final stage surgery (right) chest CT examinations. Measures for calculation of Spinal Penetration Index Surface (SPIS) and Modified Haller Index (MHI).

To evaluate the mediastinal impact, particularly with respect to the heart, the Spinal Penetration Index Surface (SPIS) was calculated, defined as the percentage of the chest cross-section occupied by the spine on an axial CT slice [19]. Higher SPIS values reflect greater intrusion into the thoracic cavity and, consequently, increased anterior-posterior compression of mediastinal structures. Although no universally accepted cutoff values exist, a SPIS between 8% and 14% is generally regarded as normal [19]. In this case, SPIS decreased from approximately 20% after the initial surgery to around 14% after the final stage, indicating reduced spinal intrusion and an increase in thoracic space (Figure 4).

Although the Modified Haller Index (MHI), defined as the ratio of the transverse chest diameter to the anteroposterior distance between the sternum and spine, is typically used for assessing pectus deformities [20], it was applied here to reflect thoracic crowding and compression. The MHI was 3.64 at the first surgery, indicating a severe pectus excavatum-like morphology (Figure 4). Following the final surgical intervention, the MHI improved to 2.79, approaching the normal range (< 2.5), consistent with a substantial reduction in anterior-posterior compression and suggestive of improved cardiopulmonary dynamics. The SPIS and MHI before and after the staged surgeries are summarized in Table 1. Thus, the overall benefit of spinal correction in this case extends beyond the restoration of spinal alignment to include thoracic expansion, which likely contributed to enhanced cardiopulmonary capacity.

**Table 1:** Spinal Penetration Index Surface (SPIS) and Modified Haller Index (MHI) after initial surgery and after final stage surgery. There are currently no universally accepted cutoff values for the SPIS; however, a SPIS of 8-14% is often considered normal [19]. For the MHI, values ≤ 2.5 are generally considered normal, and values ≥ 3.25–3.5 are often used as a surgical threshold [20].

Parameters	After initial surgery	Post final surgery
MHI	3.64	2.79
SPIS (%)	20	14

Recurrent hemodynamic instability was observed when the patient was repositioned to the prone position (Figure 2, event D). Consequently, the second stage of surgery was initiated in a right lateral oblique-prone position (Figure 3), which resulted in stabilization of hemodynamic parameters. This modified positioning was selected in view of the previously observed flattening of the left main bronchus in the fully prone position. Following unilateral screw placement and temporary rod fixation in this orientation, the spine likely achieved greater rigidity, which may account for the absence of cardiovascular instability when the patient was subsequently returned to the prone position. The increased structural stiffness probably mitigated compressive forces on mediastinal structures, thereby relieving direct cardiac compression and improving venous return. The observed clinical stabilization supports the interpretation that mechanical cardiac and bronchial compression was posture-dependent and aggravated by thoracic anatomy and intraoperative soft-tissue release. The final corrective stage was successfully completed in the prone position, which was well tolerated at that point. The use of chrome-cobalt rods, which possess greater stiffness than conventional titanium rods, likely enhanced thoracic rigidity and contributed to the uneventful completion of the procedure.

This case illustrates that modification of standard prone positioning may be beneficial in patients with predisposing factors such as mesocardia, pronounced chest wall deformity (for example, a Haller Index > 3.5), severe lordoscoliosis, or underlying connective tissue disorders. The expanding availability of navigation technologies in contemporary surgical practice facilitates such individualized positional adjustments while maintaining procedural accuracy.

## Conclusions

This case highlights the potential for cardiopulmonary compromise arising from anatomical and positional factors in patients with syndromic spinal deformities such as Coffin-Lowry syndrome and coexisting thoracic anomalies, including mesocardia, particularly during posterior spinal fusion in the prone position. Recognition of mesocardia on preoperative imaging may hold direct relevance for anesthetic and surgical planning. The observed improvement in hemodynamic stability with the use of a lateral oblique-prone position, followed by a transition to the standard prone position, suggests that a tailored approach to patient positioning can mitigate intraoperative cardiovascular risk and contribute to safer surgical outcomes.

## Figures and Tables

**Figure 5:**
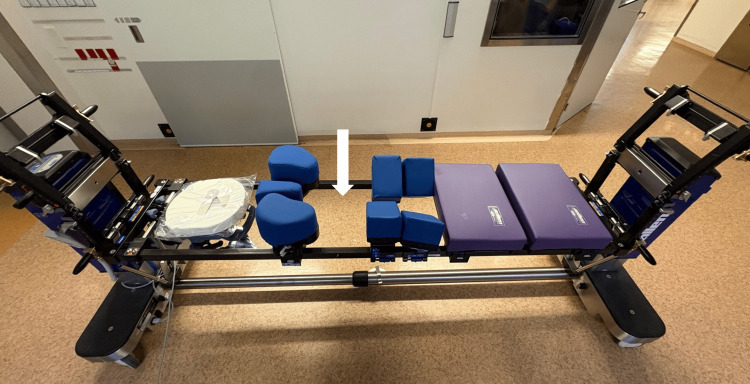
The Jackson table used during surgery supports the patient at the chest and pelvis while eliminating central support under the torso (arrow), allowing the abdomen to hang freely and reducing intra-abdominal pressure.

## Appendices

Appendix 1

**Table 2:** Intraoperative anesthesiological parameters recorded during surgery. Values represent continuous or intermittent monitoring data, including invasive and non-invasive blood pressure, heart rate, oxygen saturation, ventilatory parameters, and gas exchange. ABPs/ABPd/ABPm = arterial blood pressure (systolic/diastolic/mean, mmHg); HR = heart rate (bpm); SpO_2 _= peripheral oxygen saturation (%); NBPs/NBPd/NBPm = non-invasive blood pressure (systolic/diastolic/mean, mmHg); Puls = pulse rate (bpm); Mean airway pressure = average airway pressure (cmH_2_O); MVi = inspired minute volume (L/min); AF = airway frequency/respiratory rate (breaths/min); TVi = inspired tidal volume (mL); O_2_ insp/O_2_ exp = inspired and expired oxygen concentration (%); PEEP = positive end-expiratory pressure (cmH_2_O); CO_2_et = end-tidal carbon dioxide (kPa); I:E ratio = ratio of inspiration to expiration time (–).

Value Time	ABPs	ABPd	ABPm	HR	SpO_2_	NBPs	NBPd	NBPm	Puls	Mean airway pressure	MVi	AF	TVi	O_2_ insp	O_2_ exp	PEEP	CO_2_et kPa	I:E ratio
2025-05-20 08:00	-	-	-	-	100	-	-	-	-	2.1	10.1	2	3744.8	94.1	94.1	-	0	-
2025-05-20 08:15	-	-	-	85	100	87	53	63	-	8.9	7.0	15	470.4	76.7	74.1	5.7	6.6	0.5
2025-05-20 08:30	102	59	73	77	100	-	-	-	84	-	-	-	-	-	-	-	-	-
2025-05-20 08:45	101	62	75	86	-	-	-	-	83	11.1	7.3	15	509.6	70	68.0	5.9	4.6	0.5
2025-05-20 09:00	91	56	67	79	100	-	-	-	80	11.4	6.6	14	473.8	31.2	26.6	5.7	4.6	0.5
2025-05-20 09:15	78	50	59		100	-	-	-	76	11.5	5.5	12	462.2	30.8	25.4	5.6	4.5	0.5
2025-05-20 09:30	111	72	86	64	100	-	-	-	64	12.2	5.5	12	464.6	30.6	25.4	5.5	4.6	0.5
2025-05-20 09:45	103	67	80	64	100	-	-	-	64	12.1	5.5	12	456.8	29.2	24.1	5.1	4.6	0.5
2025-05-20 10:00	101	67	79	73	97	-	-	-	70	12.8	5.5	12	466.8	28.8	23.9	4.5	4.7	0.5
2025-05-20 10:15	104	68	80		93	-	-	-	77	13.6	5.4	12	467.6	28.6	23.5	5.7	4.9	0.5
2025-05-20 10:30	95	62	74	75	97	-	-	-	71	12.4	5.8	12	479.8	28.5	20.3	5.6	5.4	0.5
2025-05-20 10:45	87	56	67	73	100	-	-	-	73	12.4	5.9	12	491.6	29.1	22.4	5.7	5.0	0.5
2025-05-20 11:00	90	58	69	71	100	-	-	-	71	12.8	5.9	12	498.6	30.3	24.8	5.8	4.7	0.5
2025-05-20 11:15	93	59	71	71	100	-	-	-	69	12.5	5.9	12	498.6	31.1	24.4	5.3	4.8	0.5
2025-05-20 11:30	88	59	68	75	97	-	-	-	79	13.2	6.0	12	531.2	29.4	24.6	4.8	4.7	0.5
2025-05-20 11:45	104	63	75	99	87	-	-	-	83	13.6	6.0	12	522.2	28.3	21.4	5.5	5.4	0.5
2025-05-20 12:00	102	65	77	83	100	-	-	-	87	0.6	4.6	15.8	0.0	98.6	93.4	0.0	3.6	0.0
2025-05-20 12:15	-	-	-	132	100	153	100	114	-	8.1	7.3	16	457.4	98.7	93.3	3.8	5.7	0.5
2025-05-20 12:30	121	78	92	89	100	-	-	-	85	8.6	7.3	16	458.2	29.6	25.3	4.7	4.5	0.5
2025-05-20 12:45	100	55	69	94	100	-	-	-	91	8.7	7.3	16	458.4	29.4	25.1	4.5	4.3	0.5
2025-05-20 13:00	72	49	56	0	77	-	-	-	99	8.3	6.1	13	462.8	29.2	24.7	4.4	4.5	0.5
2025-05-20 13:15	101	58	72	83	100	-	-	-	77	8.2	5.9	13	455.4	74.5	70.1	4.6	4.3	0.5
2025-05-20 13:30	108	67	82	73	100	-	-	-	74	8.7	6.0	13	461.8	32.4	28.2	4.6	4.1	0.5
2025-05-20 13:45	115	71	88	64	100	-	-	-	63	8.8	6.0	13	481.4	24.3	19.6	4.5	4.2	0.5
2025-05-20 14:00	118	73	90	61	100	-	-	-	62	8.8	6.0	13	463.4	22.1	17.9	4.7	4.0	0.5
2025-05-20 14:15	119	73	91	56	100	-	-	-	56	8.9	6.0	13	463.4	25.2	20.7	4.2	3.9	0.5
2025-05-20 14:30	114	69	85	65	100	-	-	-	59	8.9	6.0	13	461.8	26.2	21.9	4.6	3.9	0.5
2025-05-20 14:45	112	67	83	56	100	-	-	-	57	8.8	6.0	13	456.8	26.3	22.0	4.3	4.0	0.5
2025-05-20 15:00	106	63	77	72	100	-	-	-	68	8.8	6.0	13	464.2	26.1	21.7	4.6	3.9	0.5
2025-05-20 15:15	99	61	74	71	100	-	-	-	60	9.0	6.0	13	469.4	26.1	21.4	4.6	4.0	0.5
2025-05-20 15:30	117	73	89	57	100	-	-	-	55	9.1	6.0	13	491.4	26.1	21.6	4.4	4.1	0.5
2025-05-20 15:45	114	73	87	64	100	-	-	-	57	8.9	6.0	13	460.8	25.9	21.7	4.3	3.9	0.5
2025-05-20 16:00	121	84	94		100	-	-	-	87	8.1	6.0	13	466.4	26.1	21.9	4.6	4.1	0.5
2025-05-20 16:15	118	74	87	73	100	-	-	-	72	8.3	6.0	13	458.2	25.8	21.4	4.5	4.2	0.5

Appendix 2
